# Hypoxia and metabolic adaptation of cancer cells

**DOI:** 10.1038/oncsis.2015.50

**Published:** 2016-01-25

**Authors:** K L Eales, K E R Hollinshead, D A Tennant

**Affiliations:** 1 Institute of Metabolism and Systems Research, College of Medical and Dental Sciences, University of Birmingham, Birmingham, UK; 2 Department of Life, Health and Environmental Sciences, University ofL'Aquila, L'Aquila, Italy

## Abstract

Low oxygen tension (hypoxia) is a pervasive physiological and pathophysiological stimulus that metazoan organisms have contended with since they evolved from their single-celled ancestors. The effect of hypoxia on a tissue can be either positive or negative, depending on the severity, duration and context. Over the long-term, hypoxia is not usually consistent with normal function and so multicellular organisms have had to evolve both systemic and cellular responses to hypoxia. Our reliance on oxygen for efficient adenosine triphosphate (ATP) generation has meant that the cellular metabolic network is particularly sensitive to alterations in oxygen tension. Metabolic changes in response to hypoxia are elicited through both direct mechanisms, such as the reduction in ATP generation by oxidative phosphorylation or inhibition of fatty-acid desaturation, and indirect mechanisms including changes in isozyme expression through hypoxia-responsive transcription factor activity. Significant regions of cancers often grow in hypoxic conditions owing to the lack of a functional vasculature. As hypoxic tumour areas contain some of the most malignant cells, it is important that we understand the role metabolism has in keeping these cells alive. This review will outline our current understanding of many of the hypoxia-induced changes in cancer cell metabolism, how they are affected by other genetic defects often present in cancers, and how these metabolic alterations support the malignant hypoxic phenotype.

## Introduction

The evolution of our multicellular ancestors from their single-celled predecessors required the development of an ability to sense changes in oxygen tension and respond with both an acute change in cell phenotype to preserve survival, but also a more long-term rearrangement of the surrounding architecture to allow better oxygen perfusion. The diffusion limit for oxygen is ~100–200 μm, which means that for adequate oxygenation, cells must be within this radius.^1, 2^ However, hypoxia is not a binary stimulus, and gradients between one functional blood vessel and the next often allow for appropriate cell and tissue development and function. During fetal development, hypoxia represents a positive, necessary stimulus, required for the appropriate patterning and function of most organs.^3^ Indeed, in some organs, a gradient in the oxygen tension across the tissue is required throughout life for their function—an example being in liver zonation.^4^ However, it is becoming increasingly clear that the cellular effects of exposure to low-oxygen tensions represent a pernicious facet of many diseases, such as cancer, cardiovascular disease, dementia and diabetes.

## Hypoxia in cancer

Hypoxia arises in tumours through the uncontrolled oncogene-driven proliferation of cancer cells in the absence of an efficient vascular bed. Owing to the rapid proliferation of cancer cells, the tumour quickly exhausts the nutrient and oxygen supply from the normal vasculature, and becomes hypoxic. This drives upregulation of the production of angiogenic factors from the hypoxic tumour sites,^5^ which triggers the vascularization of the tumour mass, a phenomenon that was first reported in 1908.^6^ However, the vessels formed in tumours are not associated with the same careful co-ordination of pro- and anti-angiogenic factors as with normal physiological angiogenesis, and lead to vascular leakiness, chaotic architecture and non-laminar blood flow.^2^ The resulting vessels are therefore not always functional, being either blunt ended or subject to changes in direction and velocity of flow. Finally, although the endothelial cells in normal vessels create a smooth, cobblestone-like surface that permits laminar non-thrombogenic flow, endothelial cells of tumour-associated vessels have gaps between them, resulting in non-laminar flow that makes the blood prone to clotting, and local tissue oedema.^7, 8^ Both these outcomes result in additional hypoxic tumour regions. As such, a solid tumour is riddled with areas of mild-hypoxia leading to severe hypoxia and necrosis as well areas of acute hypoxia and re-oxygenation.^5, 9, 10^ The chaotic architecture of the tumour vasculature can also result in dynamic fluctuations in blood flow and therefore oxygen availability, which have been observed in distinctive patterns and represent a phenomenon described as ‘cycling hypoxia'. The frequency of these cycles has been shown to vary between seconds to hours and even days.^10, 11^ The higher frequency cycling is believed to arise from the alterations in red blood cell flux and perfusion, whereas large-scale remodelling of the vascular network and angiogenesis are believed to cause the lower frequency cycling hypoxia witnessed over a matter of days.^10^ The diversity of hypoxic stimuli within tumours makes it difficult to generalize on its effect on tumour biology, and pertinent to this review, its metabolism. Indeed, the biology of a hypoxic cancer cell is a product of the interplay between the prevailing oxygen tension, hypoxia-induced signalling (often via hypoxia-inducible factor; HIF), interacting genetic defects, and cellular damage by reactive oxygen species (ROS).

## HIFs

Reductions in oxygen tension result in the stabilization and activity of the HIFs through the inactivation of a family of HIF prolyl hydroxylases (PHDs).^12, 13^ The HIF transcription factors are composed of a stable β subunit, and one of two oxygen-labile α subunits (HIF1α and HIF2α), the stability of the latter being controlled through hydroxylation by PHDs and subsequent binding and ubiquitylation by pVHL.^14, 15, 16, 17^ This leads to the rapid degradation of the α subunits in normoxic conditions through proteasomal activity. Further control is exerted through the oxygen-dependent hydroxylation of a C-terminal asparagine residue (N803 in human HIF1α) by factor inhibiting HIF1 (FIH).^18, 19^ This modification results in the inability of HIF1 to transactivate a subset of HIF target genes.^20^ Importantly, the oxygen tension required to inactivate FIH is lower than that of the PHDs,^21^ creating a graded transcriptional response appropriate to the severity of hypoxia. To transactivate target genes, the HIF transcription factors bind hypoxia-responsive elements that can be either proximal or distal to the promoter of the target genes.^22, 23^ Through this, they regulate the expression of a significant number of gene targets involved in angiogenesis, metabolic adaptation, survival and migration.^24, 25, 26, 27^ The two HIFα subunits have differential expression profiles and gene targets, providing a differential response between different tumour types. Although HIF1α is expressed ubiquitously, HIF2α expression is more restricted, and has been described in cell types such as hepatocytes and endothelial cells.^28^ The HIF-responsive transcriptome therefore varies between cell type, based on the expression profile of the α subunits, as well as the severity of hypoxia. However, it has been shown that HIF1 activity can upregulate almost all enzymes of glycolysis, facilitating increased flux in hypoxia in most if not all cell types.^29^ Importantly, in most cell types studied in chronic hypoxia, HIF1α subunit levels are rapidly increased and stabilized within hours, but after a few days decrease to a lower expression level.^30^ This is likely due to the HIF1-mediated upregulation of PHD2 (and PHD3), which appear to retain enough activity to hydroxylate HIF1α, resulting in its renewed degradation.^30, 31^ In contrast, cycling hypoxia results in an enhanced activity and stabilization of HIF1, which is at a much greater level than witnessed in chronic hypoxia.^11^ This damaging phenotype is also associated with an increased resistance to radiotherapy and chemotherapy as well as increased metastatic potential.^11^

Through the activity of HIF, cancer cells acquire many malignant properties. It is hardly surprising therefore, that tumours exhibiting significant hypoxia (intratumoural pO_2_<10 mm Hg) have been associated with an increased risk of mortality, independently of prognostic factors such as grade, histology, nodal status and size of the tumour.^32^ Primary tumour biopsies from numerous cancers such as breast, lung and pancreas have all been shown to exhibit increased expression of HIF1α or HIF2α, correlating with increased risk of metastasis and mortality.^33, 34^ Despite the overwhelming evidence in most tumours for the negative prognostic value of HIF and hypoxia in the majority of cancers, there appears to be some context dependency, as some studies have shown no prognostic significance of HIF1α.^35, 36^

## Interplay between HIF, p53 and MYC in hypoxia

**Figure 1:**
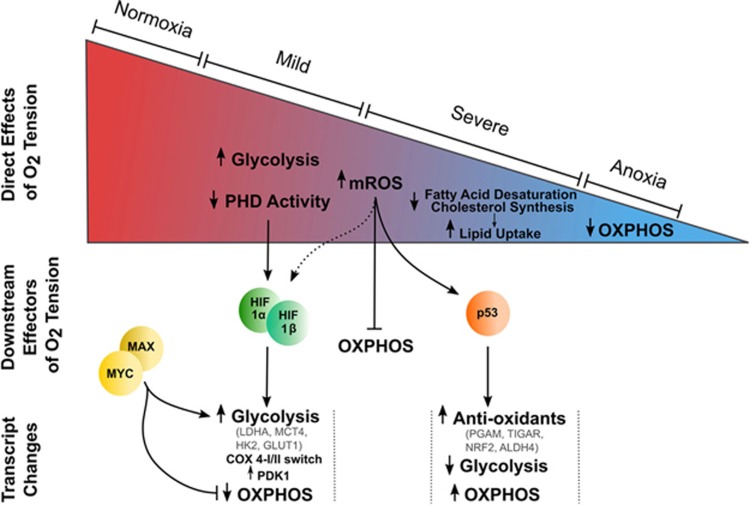
Decreasing oxygen tension elicits alterations in metabolism through a number of mechanisms. The direct effects of oxygen tension on metabolism are shown within the ‘oxygenation wedge', whereas those that are affected through signalling pathways activated in hypoxia are shown below. ALDH4, aldehyde dehydrogenase 4; GLUT1, facultative glucose transporter 1; HIF, hypoxia-inducible factor; HK2, hexokinase 2; LDHA, lactate dehydrogenase A; MAX, Myc-associated protein X; MCT4, monocarboxylate transporter 4; mROS, mitochondrial reactive oxygen species; MYC, V-Myc Avian Myelocytomatosis Viral Oncogene Homologue; NRF2, nuclear factor erythroid 2-related factor 2; OXPHOS, oxidative phosphorylation; PDK1, pyruvate dehydrogenase kinase 1; PGAM, phosphoglycerate mutase.

Hypoxia, and the HIF transcription factors have also been shown to affect the function and stability of some oncogenes and tumour suppressor genes that influence cell metabolism: the best characterized examples being v-myc avian myelocytomatosis viral oncogene homolog (MYC) and p53. The relationship between p53 and HIF is not straight forward: hypoxia has been shown to induce p53 stability in some conditions, whereas not in others, and the mechanism by which this occurs is unclear.^37, 38^ It appears that severity and duration of the hypoxic stimulus is likely to have a role, with more severe oxygen tensions eliciting a strong stabilization, perhaps through DNA damage–response mechanisms.^39^ This interplay between p53 and HIF1 is thought to result in a further gradation of the hypoxia-induced change in metabolism that is therefore dependent on HIFs during mild or acute hypoxic challenges, but engages p53-mediated gene expression changes during chronic and/or severe hypoxia (Figure 1). This could be particularly important in the control of antioxidant-producing pathways from glycolysis, which will be discussed below. In addition, the HIF transcription factors and MYC also interact to alter the MYC transcriptional profile. MYC, as a heterodimer with MYC-associated protein X (MAX), binds E-boxes in the promoter regions of genes to transactivate its targets.^40^ MAX itself is controlled through binding of MAX dimerization protein (MXD1/MAD) and MAX interactor 1 (MXI1/MAD2).^41^ Although the MYC:MAX dimer transactivates target genes, MAX binding by MXI1 inhibits this. Interestingly, HIF1α can interfere with the MYC:MAX dimer in two ways: through upregulation of MXI1 (thereby increasing competition for MAX)^42^ and through direct binding of MAX, displacing MYC. Conversely, HIF2α can bind and stabilize the MYC:MAX heterodimer, promoting MYC-induced transcriptional changes.^43^ This is more likely to be of importance in the hypoxia-induced metabolic transformation in non-MYC-amplified tumours, as the HIFα can be outcompeted in tumours with high levels of MYC expression for the MAX subunit.^44^ The stabilization of HIF1, HIF2 and p53 and the expression of MYC therefore combine to form a graded response to changes in oxygen tension with different players influencing metabolism as the degree of hypoxia increases (Figure 1).

## Hypoxia-induced changes in glucose fate and control of cellular redox

**Figure 2:**
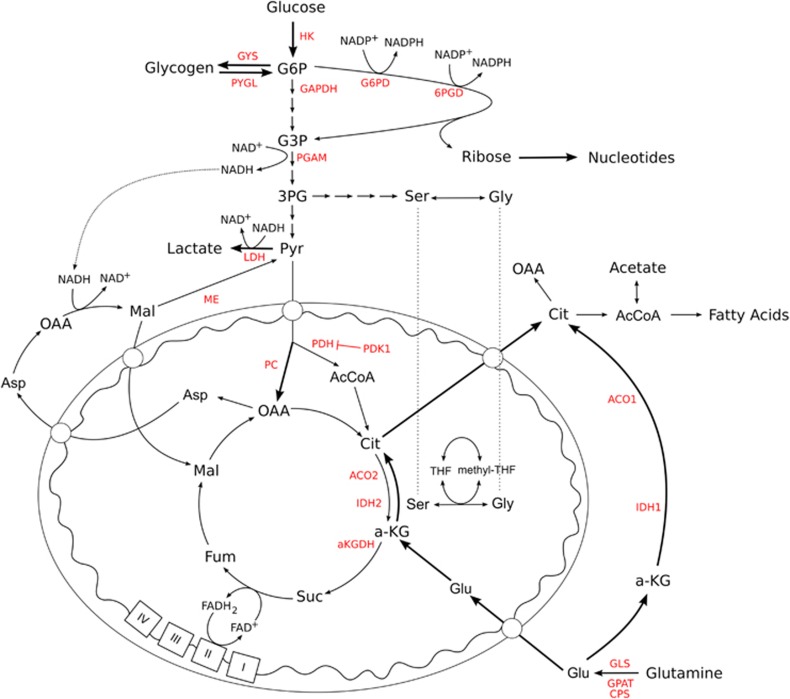
Some of the metabolic pathways known to have altered flux or importance in hypoxia. Metabolic pathways described in the text are shown, as are the enzymes mentioned, shown in red. Thicker lines represent those pathways in which hypoxic cells have been shown to increase flux, or rely more on their activity. αKG, alpha-ketoglutarate; 3PG, 3-phosphoglycerate; 6PGD, 6-phosphogluconate dehydrogenase; AcCoA, acetyl-Coenzyme A; ACO1/2, aconitase 1/2; ALD, aldolase; Asp, aspartate; Cit, citrate; CPS, cytidine triphosphate synthetase; Fum, fumarate; G3P, glyceraldehyde 3-phosphate; G6P, glucose 6-phosphate; G6PD, glucose 6-phosphate dehydrogenase; GAPDH, glyceraldehyde 3-phosphate dehydrogenase; Glu, glutamate; GLS, glutaminase; Gly, glycine; GPAT, glutamine phosphoribosylpyrophosphate amidotransferase; GYS, glycogen synthase; HK, hexokinase; IDH1/2, isocitrate dehydrogenase 1/2; LDH, lactate dehydrogenase; Mal, malate; ME, malic enzyme; OAA, oxaloacetate; PC, pyruvate carboxylase; PDH, pyruvate dehydrogenase; PDK1, pyruvate dehydrogenase kinase 1; PGAM, phosphoglycerate mutase; PYGL, glycogen phosphorylase; Pyr, pyruvate; Ser, serine; Suc, succinate.

It has been long appreciated that under hypoxic conditions glycolytic rates are enhanced, with a resulting increase in lactate production (Figures 1 and 2). Even small reductions in the production of adenosine triphosphate (ATP) by oxidative phosphorylation requires a significant increase in the rate of the ATP-producing steps in glycolysis due to the relative molar efficiencies of these processes (that is, glycolysis produces 2 mol ATP/mol glucose, whereas the addition of oxidative phosphorylation produces around 36 mol ATP/mol glucose). Although hypoxia often leads to a reduction or cessation of proliferation through HIF-mediated upregulation of p21^WAF1/CIP1^, in some cancers, proliferation is maintained through the sustained activity of mTOR or Notch.^45, 46, 47, 48^ In these cases, intracellular glucose must not only maintain cellular ATP steady-state, but also supply biosynthetic building blocks such as ribose-PP and one-carbon units for nucleotide synthesis and amino acids for protein production, putting significant pressure on glucose supply.

What has become clear recently is that cancer cells can cover for acute loss of glucose availability through the utilization of intracellular glycogen as a means of maintaining cell viability and proliferation (Figure 2).^49^ Indeed, it was recently shown that in response to acute hypoxia, cancer cells can increase their glycogen storage.^50^ In addition, the same study showed that the glycogen metabolism via the liver form glycogen phosphorylase, appeared to represent an obligate metabolic pathway for cancer cells to avoid senescence through the suppression of ROS production.^51^ This is consistent with the previously described glycogen shunt in a number of other cell types,^52^ and opens a new door to potential therapeutic opportunities.^53^

Hypoxia directly increases lactate production and excretion due to the effect of the changes in mitochondrial redox status elicited by reduced oxygen availability. Under hypoxia, the nicotinamide adenine dinucleotide reduced: oxidized (NADH:NAD^+^) ratio in the mitochondria often increases owing to slowing of electron transport and consequent reduction in the rate of NADH oxidation.^54, 55^ This inhibits NADH-producing reactions in the tricarboxylic acid cycle, thereby reducing the rate at which the malate-aspartate shuttle can transfer the NADH produced in glycolysis into the mitochondrial matrix (Figure 2). In order to maintain a favourable glycolytic rate, and therefore glycolytic ATP production, pyruvate is used to oxidize the NADH, and is consequently reduced to lactate. As cytosolic acidification also inhibits glycolysis, lactate is excreted from the cell through monocarboxylate transporters, resulting in extracellular acidification that contributes to the malignant phenotype of the cancer.^50, 56, 57^ The enzyme that reduces pyruvate, known as lactate dehydrogenase (LDH), is a pentameric complex consisting of variable ratios of the A and B subunit.^58^ The resulting isozymes have differing favoured direction of reaction, from LDH1 (5 × LDHB) that is inhibited by pyruvate and preferentially oxidizes lactate, to LDH5 (5 × LDHA), which has a higher V_max_ and favours pyruvate reduction.^59, 60^ Importantly, the hypoxia-induced alteration in the metabolic fate of pyruvate is supported by the upregulation of LDHA through the activity of HIF1, which results in a majority of LDHA subunit-containing isozymes, therefore favouring pyruvate reduction in hypoxia.^59, 60, 61^ Indeed, the stabilization and activity of HIF1 in hypoxia strongly supports and even enhances the metabolic reprogramming of glycolysis through the upregulation of almost all glycolytic genes and the monocarboxylate transporters that export lactate. HIF1 has been shown to upregulate the expression of genes encoding glucose transporters 1 and 3, glycolytic enzymes such as hexokinase 1 and 3, aldolase A and C and glyceraldehyde 3-phosphate dehydrogenase.^62^ As well as altering glycolytic isozyme selection and expression, HIF1 also modulates the function of the electron transport chain in hypoxia through switching subunits in complex IV, known as cytochrome c oxidase (COX). Under aerobic conditions, cells express the COX4-1-regulatory subunit within the COX complex. In hypoxia, HIF1 increases the expression of the alternative COX4-2 subunit, while increased expression of the mitochondrial LON protease leads to the degradation of the COX4-1 subunit.^63, 64^ This shift in subunit expression has been suggested to aid efficiency of complex IV under reduced oxygen conditions and to prevent inefficient electron transfer and potential generation of ROS in hypoxia.

Alongside the NAD^+^:NADH redox pair used to generate ATP, the other major pyridine-based redox pair is NADP^+^:NADPH, present in lower absolute quantities in most cell types. However, despite this low abundance, this latter redox pair is critical in maintaining both cellular ROS-detoxification mechanisms through reduction of the cellular redox couples such as the glutathione system, and other key metabolic pathways such as the fatty-acid synthesis pathway. As reducing equivalents cannot directly cross the mitochondrial membrane, cycling systems such as the malate-aspartate shuttle described above are used, without which the reducing potential has to be generated in the compartment where it is to be utilized. Importantly, both the oxidative pentose phosphate pathway and folate pathway have been shown to be major contributors to cytosolic NADPH production in normoxia (Figure 2).^65, 66^ Both of these pathways branch from glycolysis, and although almost all carbons (5 of 6) from the oxidative pentose phosphate pathway can re-enter glycolysis after use as reducing potential for two NADPH production, the production of NADPH through the metabolism of serine to glycine removes carbons from glycolytic ATP production (Figure 2). Interestingly, the folate pathway can also cycle reducing potential between the mitochondria and cytosol through the transport of serine and glycine across the mitochondrial membrane, due to the presence of mitochondrial and cytosolic forms of the enzymes involved (Figure 2). The resulting movement of NADPH into or out of the mitochondria could therefore be an important redox cycle in hypoxia. However, this is yet to be comprehensively investigated. What is now understood is that p53 stabilization can also regulate the fate of carbons in glycolysis by increasing the entry of glycolytic intermediates into the pentose phosphate pathway and folate pathway through the modulation of the expression of key enzymes (for example, TP53-inducible glycolysis and apoptosis regulator and phosphoglycerate mutase.^67, 68^ Through these mechanisms, p53 stabilization can upregulate antioxidant production, although this comes at the expense of glycolytic ATP production. Importantly, as mitochondrial ROS production is thought to increase in hypoxia, significant capability in generating the glutathione and thioredoxin redox couples in the mitochondria is required to maintain genetic stability, protein function, lipid membrane fluidity and therefore cell viability. The mitochondrial nicotinamide nucleotide transhydrogenase (NNT) may be critical to this, owing to its ability to transfer reducing potential from NAD^+^ to NADP^+^ and *vice versa*. The increased mitochondrial NADH:NAD^+^ ratio in hypoxia may therefore suppress the movement of ROS away from their sites of production through the maintenance of reduced mitochondrial redox couples.

Another key enzyme upregulated by hypoxia-induced HIF1 activity and alters pyruvate metabolism is the kinase, pyruvate dehydrogenase kinase 1. This HIF1-mediated effect leads to inactivation of the pyruvate dehydrogenase complex and subsequent loss of pyruvate oxidation. However, as HIF1 is upregulated in most cells significantly before oxygen becomes limiting for oxidative phosphorylation (maximal HIF stability is thought to be ~1% O_2,_ whereas respiration becomes proportional to oxygen tension ~0.4–0.7% O_2_^69, 70^), it may appear at first glance that cells react too sensitively to what could be thought of as a mild hypoxic stimulus. However, the inhibition of the pyruvate dehydrogenase complex in hypoxia could well be a protective mechanism, as it has recently been shown that activation of this enzyme complex by oncogenes is a key driver of oncogene-induced senescence through increased oxygen consumption and redox stress.^71^ This is surprisingly similar to the phenotype shown in response to the inhibition of glycogen metabolism,^51^ and points to the intriguing possibility of inducing oncogene-induced senescence in cancers through the inhibition of one or more obligate glucose-metabolising pathways.

An alternative use of pyruvate in hypoxia requires its carboxylation by pyruvate carboxylase, an enzyme traditionally associated with gluconeogenesis. This reaction has previously been shown as important for the proliferation of cancer cells in glutamine-depleted conditions in normoxia.^72^ In addition, it was recently shown to be critical for the proliferation of cells with mitochondrial defects, such as loss of fumarate hydratase or succinate dehydrogenase activities.^73, 74, 75^ It is therefore likely that cells under hypoxia will demonstrate a requirement for pyruvate carboxylase activity to re-fill tricarboxylic acid cycle carbons, permitting the synthesis of anabolic building blocks such as aspartate.

## Hypoxia-induced changes in glutamine fate in cancer

Glutamine, the most abundant non-essential amino acid in blood, is central to the anabolism of most cells in normoxia, and its uptake exceeds that of any other amino acid around tenfold.^76^ Under normoxic conditions, glutamine is oxidized to provide both ATP through the tricarboxylic acid cycle and anabolic building blocks for cell proliferation through fatty acid, amino acid and nucleotide synthesis. However, a consequence of decreased pyruvate oxidation and mitochondrial respiration during the cellular adaptation to hypoxia is increased dependence upon reductive glutamine flux for cell proliferation and viability.

Amplification of the oncogene MYC, which is observed in many different cancers, has an important role governing the rate and pathway by which glutamine is metabolized.^77^ MYC directly upregulates glutamine-metabolising enzymes such as glutaminase, which ensures the rapid integration of both the nitrogens and carbons from glutamine into the anabolic network. Interestingly, hypoxic cells with high MYC expression have increased oxidative metabolism of glutamine compared with cells with normal MYC expression.^78^ This would actively support, and promote a proliferative phenotype in hypoxic cells, making these tumours more aggressive. In addition, it is likely to also increase hypoxic ROS production, increasing genetic instability. However, this effect may be offset by the increased glutathione synthesis also observed in MYC-amplified cells.^79^ The negative effect of increased oxidative flux in MYC-amplified tumours in hypoxia would increase oxygen use in oxygen-limited conditions. This would therefore not only inhibit other oxygen-dependent metabolic reactions, but also expand areas of hypoxia and necrosis resulting in the malignant changes associated with this. It is also important to mention here that as p53-expression is important for the assembly and therefore function of COX in the electron transport chain through expression of the assembly factor, SCO2, loss of p53 in normoxia results in a similar phenotype to that observed in hypoxia as the malate-aspartate shuttle ceases to function effectively.^80^

In cells with defective mitochondria, or those in hypoxia, glutamine oxidation is decreased.^79, 81, 82, 83^ As a result, reductive glutamine metabolism has been proposed to occur in response to increases in the alpha-ketoglutarate (αKG)/citrate ratio and has since been implicated as an important pathway for the survival of these cells. Reductive carboxylation describes the synthesis of citrate using the reducing potential of NADPH via the enzymes isocitrate dehydrogenase (IDH) 1 and 2, and aconitase (ACO) 1 and 2 (Figure 2). Notably, IDH1 and ACO1 are cytosolic enzymes, whereas IDH2 and ACO2 are mitochondrial, forming two pathways with the same activity but distinct localization. IDH-mediated reductive carboxylation of glutamine-derived αKG to produce sufficient citrate for lipid synthesis was first described in normal brown adipocytes^84^ and is observed when steady-state αKG levels are high, and that of citrate is low.^85, 86^ The conditions required for a shift away from oxidative glutamine metabolism and an increase in reductive carboxylation during hypoxia are still unclear. It has been suggested that stabilization of HIF1 promotes seven in absentia homologue 2-targeted ubiquitination and proteolysis of the E1 subunit of the α-ketoglutarate dehydrogenase complex, resulting in reduced α-ketoglutarate dehydrogenase activity, decreased glutamine oxidation and therefore increased glutamine-dependent lipid synthesis, which is necessary for hypoxic cell proliferation.^87^ However, this is not supported by studies that suggest a requirement for α-ketoglutarate dehydrogenase and NNT activity for reductive carboxylation in cells either in normoxia^86^ or with mitochondrial defects, such as fumarate hydratase or complex III deficiency.^88^ Interestingly, this latter study again highlights the important role for the NNT, which has been suggested to allow the transfer of reducing potential from NADH produced by α-ketoglutarate dehydrogenase to NADPH that can be used to drive the IDH-mediated reaction.^88^ The discrepancies between these studies may lie in the nature of the perturbation: the former study investigated *de facto* hypoxia with its downstream HIF-mediated signalling, whereas the latter studies observed cells under normoxia: either wild-type, or with mitochondrial defects.

This may highlight the importance of cellular redox status in the control of mitochondrial functions, including reductive carboxylation. This is particularly apparent with respect to the ACO enzymes. Mitochondrial ACO has long been known for its susceptibility to ROS-mediated inactivation through oxidation of its Fe-S active site.^89^ In these conditions, when the pool of active ACO2 is significantly reduced, the use of IDH2-mediated mitochondrial pathway for reductive carboxylation would be less efficient, which is consistent with the findings from Metallo *et al.*,^90^ although the specific contributions of IDH1&2 (and therefore ACO1&2) in reductive carboxylation are still not entirely clear. The cytosolic form of ACO is also of interest, as it has a key role in the regulation of iron-uptake in the absence of iron, or after oxidation of its Fe-S cluster. In these situations, it has a non-catalytic role as the iron-sensing post-transcriptional regulator of mRNA translation, iron-regulatory protein 1.^91, 92, 93^ This protein binds to iron-regulatory elements in the 5' region of a number of mRNAs including transferrin and ferritin, inducing increased uptake of extracellular iron.^94, 95^

Despite decreased mitochondrial respiration and increased activity of reductive carboxylation, hypoxic cells can maintain and in some cases even upregulate oxidative glutamine metabolism, accounting for the majority of ATP synthesis through oxidative phosphorylation in these conditions.^78, 90, 96^ It is thought that this activity also facilitates the production of mitochondrial NADPH through the activity of malic enzyme (ME), which converts malate to pyruvate, and is found to be expressed at high levels in some tumours.^97, 98^ Although this is likely an important source of mitochondrial NADPH in some circumstances, it is not clear what additional benefit mitochondrial ME activity provides in the presence of an active NNT. Instead, the reduction of malate to pyruvate in the cytosol by ME1, perhaps as part of a malate-pyruvate shuttle in hypoxia (using pyruvate carboxylase), may provide a cytosolic source of NADPH.^65^ Interestingly, in normoxia, neither ME, IDH1&2 nor NNT were shown to be major NADPH sources by small interfering RNA knockdown.^65^ However, as genome-scale flux balance analysis by the same authors predicted a significant role for malic enzyme, it is likely that other pathways were capable of compensating in its absence.^65^

## ‘Alternative' carbon sources in hypoxia

The metabolism of both glucose and glutamine are progressively altered with decreasing oxygen tension. It is thought that the reduction in oxygen use before oxygen becomes limiting for oxidative ATP production may not only reduce the potential for ROS production, but also may spare oxygen for other cellular processes. There is now increasing evidence that in the absence of fully functional metabolic pathways in hypoxia, cancer cells harness exogenous and endogenous carbon sources other than glucose and glutamine to supply required metabolic intermediates.

The inhibition of pyruvate oxidation leads to a loss of mitochondrial acetyl-CoA production from glucose, and a requirement for another source of this important high-energy metabolite to continue a complete tricarboxylic acid cycle. As previously described, an alternative source of this is through the reductive carboxylation of glutamine in hypoxia. However, in addition to becoming more reliant on reductive glutamine metabolism for lipogenic acetyl-CoA production,^82^ hypoxic cancer cells are also able to metabolize exogenous acetate to derive sufficient acetyl-CoA for lipid biomass production.^99^ Indeed, the majority of hypoxic acetyl-CoA production in the latter study was shown to derive from acetate, not glutamine.^99^ Acetyl-CoA synthetase, the enzyme responsible for this reaction, is found to be critical for hypoxic cancer cell survival, and its expression is induced in response to hypoxia for lipid synthesis.^100^ Changes in the availability of acetyl-CoA in the cytosol and mitochondria under hypoxia are likely to alter the acetylation of target proteins in these compartments, which include a number of key metabolic enzymes such as glutamate dehydrogenase and mitochondrial superoxide dismutase.^101^ It is likely that mitochondrial acetyl-CoA concentrations decrease in hypoxia due to the inhibition of pyruvate dehydrogenase complex activity, but at present this is unknown.

There is also limited data on the effect of hypoxia-induced autophagy on cancer cell metabolism. Although it is accepted that damaged organelles, especially mitochondria, are cleared through autophagy under hypoxia, the fate of the nutrients released from these organelles is currently unknown. However, a metabolomics profile consistent with nutrient release due to autophagy has been reported.^102^ It is likely that the nutrients released from processes such as mitophagy are used to support ongoing cell viability and repair of cellular structures such as DNA in hypoxic cancer cells. Indeed this is somewhat supported by the limited evidence available that shows a drop in ATP and increase in cell death upon inhibition of autophagy in hypoxia.^102^

Interestingly, it was recently shown that Ras-transformation of cells resulted in an increase in macropinocytosis—a process by which cells engulf extracellular fluid and its contents—to supply amino acids for metabolism in normoxia.^103^ Although macropinocytosis has not formally been shown to be induced in hypoxic cancer cells, a recent study found that uptake of unsaturated lipids (lysophospholipids) increased in hypoxic cancer cells, and that this was phenocopied in normoxia by Ras-transformation.^104^ Hypoxic cancer cells may have difficulty in synthesising the correct species of lipids for appropriately functional membranes at a rate that would keep up with their oncogenic drive, due to hypoxia-mediated loss of glucose oxidation and inhibition of the oxygen-dependent desaturation of fatty acids (stearoyl-coA desaturase; SCD1). Uptake of exogenous unsaturated fatty acids, similar to the Ras-induced macropinocytosis for amino acid supplementation, allows them to maintain rapid proliferative rates despite a hostile microenvironment.

## Metabolic complementation

Most tumours are characterized by regions of necrosis, mild and severe hypoxia and normoxia. As such, in this heterogeneous environment, there are significant opportunities for metabolic complementation between different microenvironments and cell types. An example of the latter is the oxygen-sparing activity of tumoural vascular endothelial cells, which, as the cells most proximal to the blood supply, are highly glycolytic.^105^ This permits oxygen to permeate further into the tumour, alongside the remaining glucose and other nutrients that can be oxidatively metabolized. However, an interesting and potentially very important metabolic interplay between hypoxic and normoxic tumour areas was revealed by Sonveaux *et al.*^106^ in 2008. The authors showed that much like some other tissues, normoxic tumour areas can oxidize lactate as a significant carbon source, sparing glucose and allowing it to diffuse further away from the tumour vasculature.^106^ Indeed, upon reaching the hypoxic tumour areas, anaerobic metabolism was used to metabolize the glucose to lactate, which could then be used by the normoxic tumour areas. This metabolic symbiosis is now considered a strong therapeutic target, most likely through the inhibition of one or more of the monocarboxylate transporters required to transport lactate in and out of the cell.^107^

## Conclusions

Although our knowledge of metabolic transformation in cancer has improved markedly over the past few years, the impact of hypoxia on most cellular metabolic pathways is still not entirely clear. Hypoxia-induced metabolic re-wiring is designed to permit cell and tissue survival during the metabolic stress. For some diseases we need to support these changes in order to preserve tissue function. However, hypoxia drives malignant progression in cancers, resulting in poorer survival through resistance to therapy and increased metastatic potential. We therefore need to understand how hypoxia alters cellular metabolism in order to be able to target these pathways, thereby killing these malignant cells. Targeting those pathways that are merely more highly utilized in hypoxic cancer cells, such as glycolysis, are unlikely to be clinically viable, as the therapeutic window may be difficult to achieve. However, other approaches that identify those pathways that are usually dispensable, but become fundamental to survival under hypoxia, are likely to result in therapeutic targets that have little to no side-effects to normal tissue. Although these therapies will not efficiently target the normoxic areas of tumours, they would be expected to increase the efficacy of other commonly used interventions such as radiotherapy, thereby improving the overall survival of patients.
